# Reducing sedentary behavior through workplace counseling: effects on activity, sitting breaks, and well-being – a randomized controlled trial

**DOI:** 10.1186/s13690-026-01867-6

**Published:** 2026-02-28

**Authors:** Tobias Engeroff, Larissa Kraus, Jan Wilke, Daniel Niederer, David Groneberg

**Affiliations:** 1 Institute of Occupational, Social and Environmental Medicine, Goethe University, Frankfurt/Main, Germany; 2 Department of Neuromotor Function and Movement, University Bayreuth, Bayreuth, Germany

**Keywords:** Workplace physical activity, Sedentary behavior, Individualized counseling, Hybrid work, Behavioral intervention

## Abstract

**Background:**

The increase in hybrid-work or work-from-home demands setting-specific strategies to improve health-promoting behaviors. This study compared the effects of a behavioral intervention based on individualized workplace counseling to an educational approach based on a standardized factsheet.

**Methods:**

University employees (*n* = 121) were randomly allocated to three groups receiving: (1) a factsheet visualizing the World Health Organization (WHO) guidelines on physical activity and sedentary behavior, (2) 20 min of individualized counseling tailored to individual barriers, opportunities, and personal strengths, or (3) no advice (control group). Physical activity (minutes per week) and sedentary behavior (minutes per day) during leisure- and occupational time were assessed before and 4 weeks after interventions using the Nordic Physical Activity Questionnaire. Further outcomes were well-being (WHO-5 questionnaire), pain intensity, and pain interference with work (both with the Short Form 36 Health Survey). Descriptive data are indicated as mean and standard deviation, and the effects of the interventions were analyzed using analysis of variance (ANOVA).

**Results:**

The participants (age 43.6, 12.2 years, body mass index 24.3, 3.9 kg/m²) showed high levels of leisure-time physical activity (294.30, 215.37 min) and moderate levels of sedentary behavior (504.33, 158.82 min) at baseline. Overall, physical activity levels (32.37, 112.51 min) and especially vigorous intensity levels (3.88, 19.64 min) at work were low. A large share of daily sedentary time was accumulated during work (414.05, 105.19 min). The individualized counseling group decreased workplace sedentary time (-30.73, 81.74 min), compared to the factsheet (0.00, 54.35 min) and control groups (+ 6.00, 72.92 min), and increased workplace vigorous intensity activity (+ 10.24, 43.10 min), compared to the factsheet (+ 0.50, 3.16 min) and control group (-6.75, 28.77 min) (*p*<.05). No intervention showed any effect on leisure activity levels, well-being or perceived bodily pain (*p*>.05).

**Conclusion:**

An individualized approach to setting-specific physical activity counseling improves health-promoting behaviors in physically active persons with sedentary occupations.

**Trial registration:**

Registered at the German Clinical Trial Register: drks.de (registration number: DRKS00031671; 04 Feb 2025). Retrospectively registered.

**Supplementary Information:**

The online version contains supplementary material available at 10.1186/s13690-026-01867-6.

**Table:** 

Text box 1. Contributions to the literature
• Demonstrates that individualized, one-time workplace counseling can significantly reduce occupational sedentary time and increase vigorous physical activity — even in already active office workers — for example, by encouraging stair use instead of elevators and promoting active lunch breaks.
• Highlights the limitations of purely educational interventions (e.g., factsheets) for behavior change in sedentary workplace settings.
• Provides new evidence on sedentary behavior patterns in hybrid and academic work environments, including work-from-home arrangements.
• Suggests that reductions in sedentary time alone may be insufficient to improve well-being or pain in highly active populations — calling for more targeted, long-term interventions.

## Background

Sedentary behavior, defined as sitting or reclining with an energy expenditure ≤ 1.5 METs [1], is recognized as an important lifestyle-related risk factor for all-cause mortality [2] and chronic diseases such as cancer, cardiovascular, and metabolic disorders [3, 4]. The World Health Organization (WHO) therefore recommends reducing or interrupting prolonged sitting [5].

On average, people spend about 9 h per day sedentary, six of which occur at work [6]. NHANES 2005/2006 data show that the proportion of sedentary time ranges from 44% to 66% depending on occupation [7]. To mitigate adverse effects, the WHO advises incorporating regular active breaks [5].

In view of the high amount of sedentary behavior, the workplace is one of the most relevant settings for interventions. Educational, behavioral, and environmental strategies have been proven effective to reach these goals [8]. Yet, the increasing flexibility of workplaces, including hybrid and remote work, has led to significant changes in workplace behavior, highlighting the relevance of approaches that do not rely on specialized equipment (e.g., adjustable workstations) or complex environmental adaptations [9]. A particular challenge in occupational health practice is limited resources, as counseling is often feasible only once within a timeframe of multiple months or years during routine preventive care. Consequently, our study applied this realistic setting of a single health counseling session to examine whether such a low-intensity intervention can effectively influence sedentary behavior.

This randomized controlled trial (1) assesses overall- and occupational sedentary behavior and physical activity in work-from-home-workers and regular workers, (2) analyzes the impact of sedentary behavior during work and leisure time on well-being and (3) compares the effect of an educational intervention (a standardized factsheet) and a combined behavioral and educational intervention (individual workplace counseling) on health enhancing physical activity, sitting time and breaks in sitting time during work. We hypothesized that participants receiving individualized counseling would show a greater reduction in sedentary time and a greater increase in physical activity compared to those receiving only an informational factsheet or no intervention (control group).

## Methods

### Study design and ethical aspects

Our study adopted a three-armed, randomized, controlled experimental design. The study was approved by the local independent ethics commission (reference number: 2021-43; 14, Aug 2021) and registered at the German Clinical Trial Register: drks.de (registration number: DRKS00031671; 04. Feb 2025). The investigation was conducted in accordance with the Declaration of Helsinki (Version Fortaleza 2013). Before participating in the study, each volunteer signed a written informed consent form.

### Participants

We recruited university employees aged 18 to 65 through direct advertising on campus. Eligible participants were primarily desk-based office workers whose roles required them to remain at their workstation for a substantial part of the day. To ensure that the intervention targeted individuals with a meaningful level of occupational sedentary behavior, participants had to report sitting for at least 4 h per workday on average. Exclusion criteria included medical conditions or physical limitations that would preclude safe participation in increased physical activity.

### Experimental setup

Participants were randomly allocated to one of three groups: two intervention groups or the control group. The randomization sequence was generated using Microsoft Excel, employing balanced block randomization (*n* = 18 per block) to ensure equal group sizes. The sequence was created by a researcher not involved in participant recruitment or data collection to maintain allocation concealment.

Participants of the two intervention groups either (1) participated in an individualized 20-minute physical activity counseling or (2) received a standardized factsheet containing guidelines on health-enhancing physical activity and sedentary behavior. The control group received no treatment. At baseline and after the 4-week intervention period, all outcomes were assessed. The intervention period of four weeks was chosen to allow sufficient time for participants to implement behavioral changes while maintaining feasibility within occupational health settings.

### Interventions

Participants in the control group did not receive any treatment but were, of course, allowed to continue any health-enhancing habits.

Participants in the factsheet intervention group received a factsheet. The factsheet (1 page, DIN A4) included a comprehensible designed and illustrated overview of the positive effects of physical activity and the application of current recommendations for health-enhancing physical activity. Supplement 1 contains the factsheet. The benefits listed included potential risk reductions for metabolic diseases, cancer, musculoskeletal health problems, and depression, and positive effects on general health, sleep, quality of life, stress, and body weight. Information on current recommendations included four different areas: (1) moderate to vigorous physical activities, including information on the volume and examples for activity types; (2) resistance exercises and strengthening activities; (3) reducing or interrupting sedentary behavior; and (4) improving balance to reduce the risk for falls, specifically for participants older than 64 years. The factsheet was distributed after the baseline assessments.

Participants in the counseling group received both the factsheet and a 20-minute consultation by a trained medical specialist. The counseling was based on the factsheet but tailored to each participant’s individual barriers, opportunities, and personal strengths to develop a realistic plan for integrating physical activity into daily life. As part of the consultant approach, the specialist considered workplace factors (e.g., work schedules, meeting patterns, opportunities for short activity breaks), health aspects (e.g., pre-existing conditions or movement limitations), and current activity habits and motivation. Suggested strategies ranged from small, practical changes—such as taking the stairs instead of the elevator, incorporating brief active breaks during work, or taking active lunch breaks—to meeting recommended activity levels, such as 30 min of moderate or 20 min of vigorous activity on most days. The counseling also took into account participants’ different work situations (e.g., work from home vs. regular office work) and prioritized recommendations according to feasibility and individual needs.

### Outcomes

During the baseline examination, participant characteristics, personal and anthropometric data, including age, sex, height, weight, and education, were assessed. Participants also reported whether they work from home on at least one day of a typical week, if they live in a rural or urban area, and about their family status, including the number of children. Finally, participants were asked about their state of health and asked to name chronic musculoskeletal, internal, or neurological diseases and, if applicable, the number of medications they were taking. Pre-intervention assessments took place after baseline examinations, and post-intervention assessments were performed after 4 weeks. Pre and post assessments included (1) Physical activity with moderate and vigorous intensity [minutes] by means of the short form of the Nordic Physical Activity Questionnaire (NPAQ) [10], (2) Well-being (% score) using the WHO-5 questionnaire [11], (3) Pain intensity and pain interference with work ranging from zero to 100 points by means of the Bodily Pain Scale of the Short Form 36 Health Survey Questionnaire (SF-36) [12] and (4) Time spend with sedentary behavior in hours per day using the short form International Physical Activity Questionnaire (IPAQ) [13]. Furthermore, the time spent sedentary during work in hours per day and the number of physically active interruptions during sedentary work were assessed using two standardized questions. For further analysis, participants were grouped based on whether they fulfill current recommendations for health-enhancing physical activity (≥150 min of moderate intensity physical activity or ≥75 min of vigorous intensity physical activity or an equivalent combination of both) [5].

### Statistical analysis

We applied Microsoft Excel (Version 16.68) for data processing, Jamovi (Version 2.3.21) for data analysis, and Prism (Version 9.5.1) for figures. Data were tested for normality using Shapiro-Wilk tests and checked for outliers using box and whisker plots. Descriptive data were reported as means and standard deviations.

Kruskal-Wallis tests were applied to analyze between-group differences at baseline and to evaluate the impact of nominal scaled lifestyle and workplace factors, including work-from-home application, smoking, health status, medication, and family status on baseline sedentary behavior and physical activity data. Spearman correlation was used to analyze associations of baseline physical activity and sedentary behavior with the number of children and the years of education.

Analyses of covariance (ANOVA) of pre to post intervention differences controlled for the baseline values were applied to analyze the main manipulation (between-groups*time) effects (Counseling versus factsheet versus control) on physical activity and sedentary behavior outcomes, as well as on well-being and bodily pain. In case of significant omnibus ANOVA testing, post hoc t-tests were conducted. For the ANOVA, eta squared (η²) was reported as an effect size measure, with η² values of approximately 0.01 interpreted as small, 0.06 as medium, and 0.14 or higher as large effects. Chi squared tests were applied to test for pre to post group differences in the number of adherers to physical activity guidelines.

## Results

### Descriptive data

**Fig. 1:**
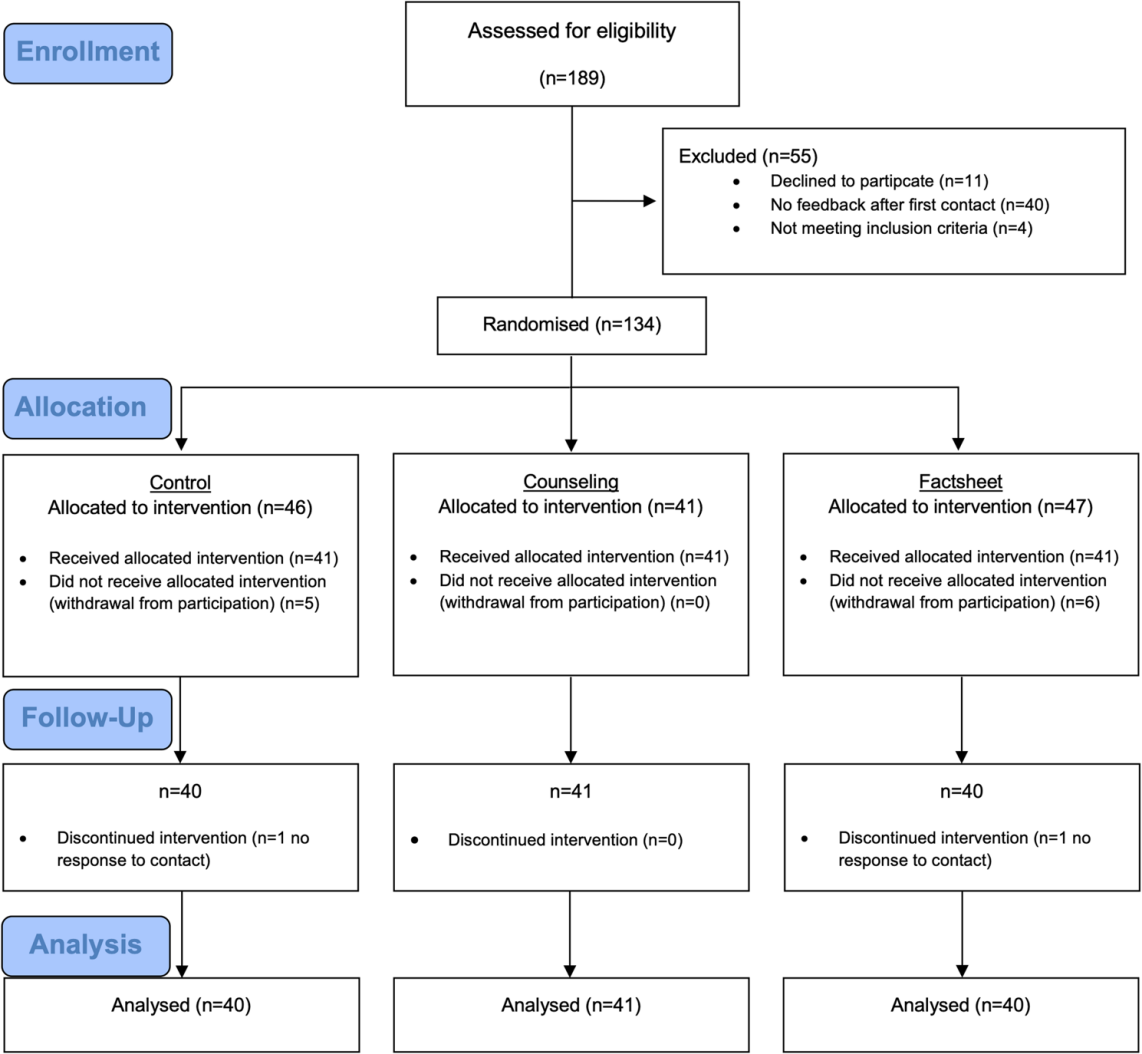
CONSORT flow diagram. One hundred thirty-four participants were assigned to the control (*n*=46), to the workplace counseling (*n*=41), or to the factsheet (*n*=47) group by randomization. CONSORT 2010, Consolidated Standards of Reporting

A total of 134 participants were included in the study and randomized in one of the intervention groups (Control *n* = 46, factsheet *n* = 47, counseling *n* = 41). Thirteen participants did not complete the intervention, and the data of the remaining 121 participants (*n* = 57 male, *n* = 64 female, *n* = 0 non-binary/diverse) were available to analyze the main manipulation effects. Study and participant flow are displayed in Fig. 1. Participant characteristics data and health status are shown in Table 1.

**Table 1:** Anthropometrical-, lifestyle- and health related data (chronic musculoskeletal, internal or neurological diseases and the number of medications) of the study collective (total sample and separated by groups); Data indicated as mean and standard deviation; Results of the between group comparisons indicated using F-values in case of ANOVA or χ^2^-values in case of chi-squared tests and *p*-values; BMI = Body-Mass-Index; cm = centimeter, kg = kilogram; m² = square meters

Outcome (Mean, Standard deviation)	total sample (*n* = 121)	Control (*n* = 40)	Counseling (*n* = 41)	Factsheet (*n* = 40)	Between group comparison (F- or χ^2^, *p*-value)
Age (Years)	43.6, 12.2	44.4, 13.3	42.3, 11.6	44.2, 11.7	0.362; 0.697
Body height (cm)	175, 9.1	175, 8.1	173, 9.3	175, 10.0	0.655; 0.522
Body weigth (kg)	74.5, 14.7	76.4, 14.5	73.3, 14.4	74.0, 15.4	0.491; 0.614
BMI (kg/m²)	24.3, 3.9	24.8, 4.4	24.3, 4.1	23.8, 3.3	0.674; 0.513
Family status (number of singles)	*n* = 32	*n* = 9	*n* = 10	*n* = 13	0.275; 0.760
Number of children	0.84, 1.02	0.88, 0.97	0.78, 0.88	0.88, 1.2	0.134; 0.875
Education years	20.0, 6.45	19.8, 7.63	20.5, 4.65	19.6, 6.86	0.275; 0.760
Work-from-home	*n* = 33	*n* = 11	*n* = 12	*n* = 10	0.188; 0.910
Smokers	*n* = 9	*n* = 5	*n* = 0	*n* = 4	5.16; 0.076
Musculoskeletal diseases	*n* = 2	*n* = 1	*n* = 0	*n* = 1	1.04; 0.594
Internal diseases	*n* = 33	*n* = 8	*n* = 14	*n* = 11	2.04; 0.360
Neurological diseases	*n* = 4	*n* = 2	*n* = 2	*n* = 0	2.04; 0.360
Diseases in multiple categories (number of categories)	*n* = 85 none	*n* = 30 none	*n* = 27 none	*n* = 28 none	1.95; 0.376
	*n = 33 one*	*n = 9 one*	*n = 12 one*	*n = 12 one*	
	*n = 3 two*	*n = 1 two*	*n = 2 two*	*n = 0 two*	
Medication	*n* = 31	*n* = 9	*n* = 13	*n* = 9	1.21; 0.547

### Baseline physical activity, sedentary time, and breaks during occupational sedentary time

Table 2 shows baseline data for physical activity and sedentary behavior. During baseline, our sample reached a mean of 1641.96 (standard deviation 1332.06) metabolic equivalent of task (MET) minutes of physical activity. Sixty-nine out of 121 participants participated in more than 150 min of moderate or 75 min of vigorous intensity physical activity per week, thus they were already fulfilling current recommendations for health-enhancing physical activity. Contrarily, most of the participants (*n* = 94) indicated not engaging in physical activity with moderate or vigorous intensity that lasted at least 10 min during work at baseline.

**Table 2:** Pre-intervention data (total sample) and the pre- to 4-week post-intervention differences for physical activity and sedentary behavior of the three intervention groups; means, standard deviation, ANCOVA, and post hoc test results; significant manipulation effects are marked with an asterisk

Outcome (Mean, Standard deviation)	Baseline	Change scores since baseline			
	(*n* = 121)	Control group (*n* = 40)	Counseling group (*n* = 41)	Factsheet group (*n* = 40)	ANCOVA (F-, *p*-value, η²)
Overall activity (minutes per week)	326.67, 259.83	28.20, 284.51	136.95, 233.36	104.50, 301.03	1.82, 0.167, 0.03
Overall sedentary time (minutes per day)	504.33, 158.82	-12.89, 120.83 Vs. counsel 0.84, 0.680	-37.21, 124.56 Vs. factsheet − 3.45, 0.002*	+ 66.22, 153.88 Vs. control 2.61, 0.028*	6.45, 0.002, 0.06*
Work activity overall (minutes per week)	32.37, 112.51	-15.05, 150.50	+ 24.63, 128.01	+ 35.00, 86.08	2.12, 0.125, 0.02
Work activity moderate (minutes per week)	28.49, 104.90	-8.30, 132.29	+ 14.39, 138.83	+ 34.50, 85.51	1.25, 0.291, 0.01
Work activity vigorous (minutes per week)	3.88, 19.64	-6.75, 28.77 vs. counsel 2.17, 0.081	+ 10.24, 43.10 vs. factsheet 2.49, 0.037*	+ 0.50, 3.16 vs. control − 0.31, 0.950	3.71, 0.027, 0.04*
Work sedentary time (minutes per day)	414.05, 105.19	+ 6.00, 72.92 Vs. counsel − 2.69, 0.022*	-30.73, 81.74 Vs. factsheet − 2.44, 0.043*	0.00, 54.35 Vs. control − 0.24, 0.968	4.42, 0.014, 0.06*
Breaks during sedentary work (number per day)	9.02, 8.87	-1.05, 12.19	+ 0.44, 8.09	+ 0.97, 7.98	1.07, 0.345, 0.01
Leisure activity overall (minutes per week)	294.30, 215.37	+ 43.25, 181.21	+ 112.32, 207.62	+ 69.50, 237.80	1.12, 0.330, 0.02
Leisure activity moderate (minutes per week)	186.20, 151.82	+ 21.75, 183.00	+ 75.24, 135.38	+ 17.5, 171.24	1.12, 0.331, 0.02
Leisure activity vigorous (minutes per week)	108.10, 138.26	+ 21.50, 120.95	+ 37.07, 166.86	+ 52.00, 167.84	0.28, 0.755, 0.01

We evaluated the impact of health, lifestyle, and workplace factors on baseline sedentary behavior and physical activity data. Participants working from home (557.58, 199.10 min per day) reported significantly (χ^2^ = 3.95, *p*=.047, ε^2^ = 0.03) more overall sedentary time compared to regular office workers (484.37, 136.88 min per day). Singles (10.97, 8.19 breaks during a workday) reported fewer (χ^2^ = 4.05, *p*=.044, ε^2^ = 0.03) breaks during sedentary work than participants in a relationship (8.84, 10.61 breaks). Furthermore, the number of children showed a negative association with overall sitting time (Spearman’s rho − 0.21, *p*=.020) and sitting time during work (Spearman’s rho − 0.20, *p*=.025). Participants with chronic diseases reported significantly less overall leisure physical activity (261.37, 181.05 min per week; χ^2^ = 4.08, *p*=.043, ε^2^ = 0.03) and moderate intensity leisure physical activity (156.13, 137.46 min; χ^2^ = 10.27, *p*=.001, ε^2^ = 0.09) compared to healthy participants (Overall: 369.05, 266.04 min; moderate: 254.46, 162.40 min). Likewise, participants on medication reported significantly (χ^2^ = 5.79, *p*=.016, ε^2^ = 0.05) lower amounts of moderate intensity leisure physical activity (168.33, 143.74 min per week) than participants without regular medication (251.30, 165.46 min). Further, analysis of anthropometrical, health, and lifestyle data including years of education showed no impact on baseline sedentary behavior or physical activity (*p*>.05).

### Intervention effects on physical activity, sedentary time, and breaks during occupational sedentary time

**Fig. 2:**
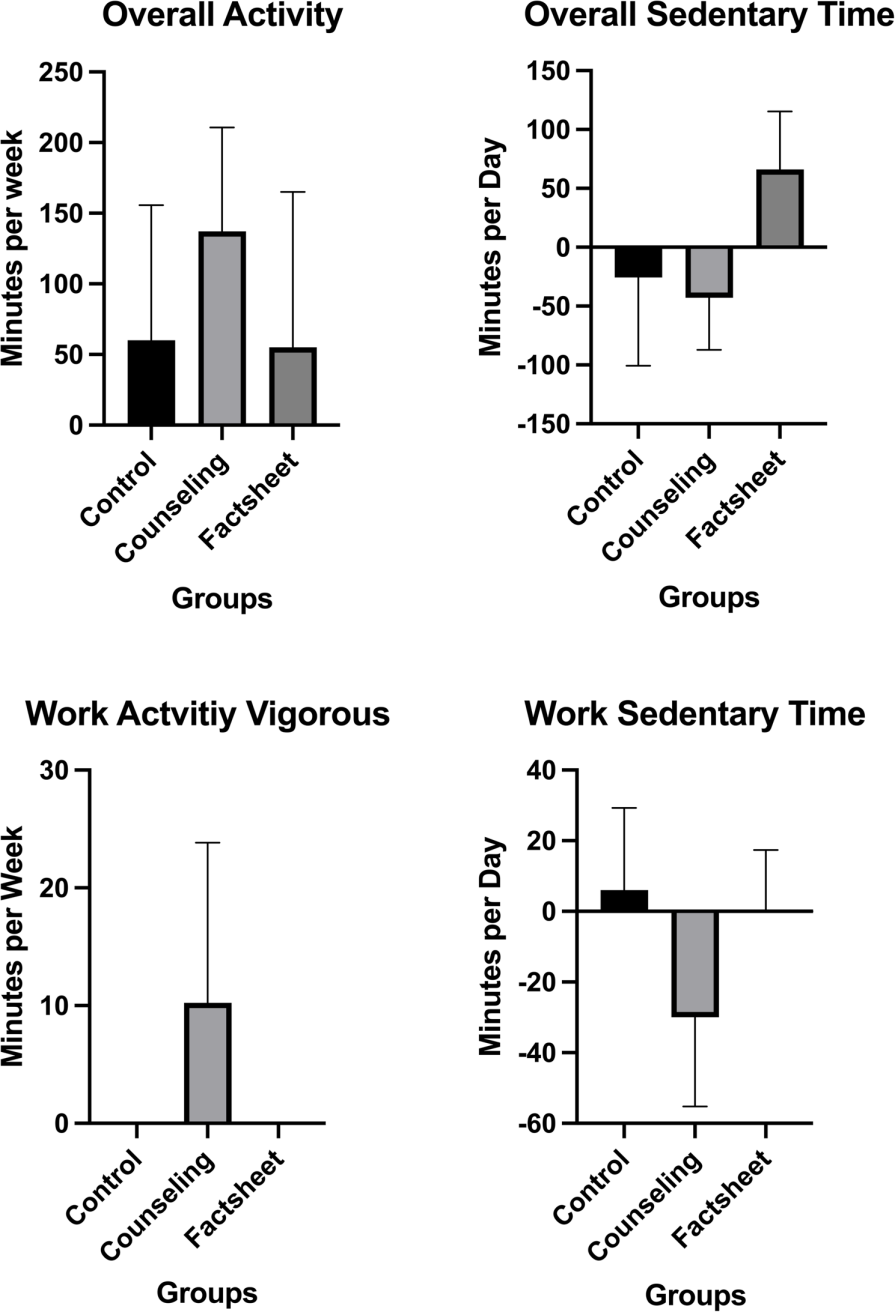
Median and interquartile range of pre- to 4-week post-intervention differences for overall physical activity and sedentary behavior, as well as for physical activity with vigorous intensity and sedentary time during work of the three intervention groups

Pre-intervention data and the pre- to 4-week post-intervention differences for physical activity and sedentary behavior are displayed in Table 2. Pre-intervention data were not significantly different between groups. The interventions showed no effect on overall physical activity levels or overall sedentary time (*p* > .05). Workplace counseling had a positive impact on the amount of vigorous physical activity during work (*p* = .027) and led to a reduction in sedentary time during work (*p* = .014). Figure 2 shows median and interquartile range for pre to post changes of overall physical activity and sedentary time, vigorous activity during work and sedentary time during work.

During baseline, the number of participants complying with physical activity guidelines was not different within the three intervention groups (Counseling: 32 yes / 9 no, factsheet: 33 yes / 7 no, control: 31 yes / 9 no; χ^2^ = 0.37, *p*=.832). After the intervention, significantly (χ^2^ = 6.47, *p*=.039) more participants of the counseling group (39 yes / 2 no) reported fulfilling current physical activity recommendations compared to the control- (34 yes / 6 no) and factsheet (30 yes / 10 no) group. Participants of the factsheet group increased daily sedentary time compared to both other groups, whilst their workplace sedentary time remained unchanged. This led to significant ANCOVA effects for overall sedentary time (*p*=.002).

### Intervention effects on well-being and bodily pain

Table 3 shows the scores achieved on the WHO-5 questionnaire and the SF-36 questionnaire subscale on bodily pain. Data are indicated as baseline and as pre- to 4-week post-intervention differences. At baseline, thirteen participants showed a WHO 5 overall raw score below thirteen (equals a percentage score below 50%), and the mean SF 36 average rating was 77%. At baseline, neither physical activity nor sedentary behavior data were associated with the WHO-5 overall raw score or the SF-36 average rating. Interventions showed no effect on pre- to post differences of the WHO-5 overall raw score and the SF-36 average ratings.

**Table 3:** Pre-intervention data (total sample) and the pre- to 4-week post-intervention differences for the WHO-5 and the SF-36 questionnaire subcategory on bodily pain; means, standard deviation, ANCOVA results

Outcome (Mean, Standard deviation)	Baseline	Change scores since baseline			
	(*n* = 121)	Control group (*n* = 40)	Counseling group (*n* = 41)	Factsheet group (*n* = 40)	ANCOVA (F-, *p*-value, η²)
WHO 5 - Overall raw score (sum)	16.78, 3.44	-0.80, 3.34	-0.34, 2.73	0.75, 3.20	2.19, 0.116, 0.03
WHO 5 - Cheerful and in good spirits	3.74, 0.82	-0.17, 0.78	-0.32, 0.85	0.17, 0.81	
WHO 5 - Calm and relaxed	3.32, 0.94	-0.07, 0.94	-0.02, 0.99	0.00, 1.06	
WHO 5 - Active and vigorous	3.31, 0.85	-0.30, 1.18	-0.17, 0.95	0.28, 1.06	
WHO 5 - Fresh and rested	2.72, 1.10	-0.25, 1.30	0.22, 0.94	0.42, 1.26	
WHO 5 - Life has been filled with interesting things	3.69, 1.01	0.00, 0.85	-0.05, 0.86	-0.13, 1.14	
SF 36 Average in percent	77.25, 24.80	1.00, 25.91	2.01, 24.52	-0.50, 21.93	0.27, 0.764, 0.00
SF 36 Bodily pain in percent	70.41, 27.70	2.00, 29.63	3.41, 27.17	-1.00, 25.20	
SF 36 Pain interference with work in percent	84.09, 25.00	0.00, 28.87	0.61, 25.91	0.00, 20.41	

## Discussion

Compared to a group receiving information on health enhancing physical activity via a factsheet and a control group, (1) the individualized counseling group showed a greater reduction in workplace sedentary time; and (2) a greater increase in workplace vigorous intensity activity. Our hypothesis can, thus, be accepted.

In contrast, neither a single 20-minute counseling session nor a factsheet on health-enhancing physical activity and sedentary behavior significantly increased overall physical activity levels or physical activity during leisure time in this already highly active sample. As a possible explanation, most of the university employees included in this study already reached or exceeded current recommendations for health-enhancing physical activity at baseline. In contrast, overall physical activity levels and especially the amount of vigorous intensity activity during work were reported to be low. Furthermore, neither well-being nor bodily pain was linked to physical activity or sedentary behavior at baseline. The reported changes in physical activity behavior showed no impact on well-being or bodily pain.

### Baseline sedentary behavior and physical activity

With a mean of 1642 MET minutes, more than half of our participants not only reached but exceeded recommended levels of physical activity at baseline, indicating a highly active sample and limiting the potential scope for further improvement through the intervention. Although these values seem high, they are in line with a representative sample of the German population based on a cross-sectional telephone survey [14]. In this survey, 72% of the included 2800 participants reported spending more than 1200 MET minutes physically active. Conversely, participants in our study spend only 30 min of physical activity per week during work, and 78% reported never reaching vigorous intensity during workplace activities. Although participants working from home reported higher sitting times, the number of home office days varied considerably, and contextual factors were not systematically assessed. Therefore, this finding should be interpreted with caution and considered a potential direction for future research. The low level of vigorous intensity physical activity among office workers had already been shown by a previous study, which reported a total of 5 to 10 min per day [15], illustrating that this is a potential target for interventions.

With 504 min overall sedentary time and a share of 414 min during work, our participants did not match the mean of 554 min reported for the German population in 2022, but exceeded the average of 240 min of sedentary time during work [14]. In line with the meta-analysis of Prince and colleagues, our data thus indicated that a large share of overall sedentary time is spent during work [6].

### Intervention effects

Our baseline data underline that the workplace is one of the most relevant settings for interventions aimed at reducing the detrimental effects of sedentary behavior, particularly by decreasing sitting time or interrupting prolonged sitting with physically active breaks. While educational strategies, such as distributing flyers or factsheets, and behavioral interventions, such as counseling, have been shown to be effective in a recent meta-analysis [8], our findings suggest that, with a single exposure approach, only the behavioral intervention can lead to a change in exercise behavior. This is consistent with findings by Purath et al. [16] who reported a significant increase in overall physical activity and time spent walking in sedentary women six weeks after a physical activity counseling. Contrastingly, in a study of Aittasalo et al. [17], participants received counseling on leisure physical activities and did not increase their habitual activities in a 6 and 12-month follow-up. In our study, a purely educational intervention, such as the one-time provision of an easy-to-understand information sheet, had no noticeable effect. One explanation for the lack of impact of the intervention could have been the rather general approach and presentation of information about the benefits and effects of physical activity. A current review concludes that educational interventions need to be customized to the environment through methods such as point-of-decision prompts to achieve a significant effect.

One of the key advantages of our behavioral approach, compared to environmental interventions or purely educational strategies, lies in the personalized nature of the counseling. By including individualized advice and implementation strategies tailored to office, hybrid, and work-from-home settings, we were able to foster health-promoting behavioral changes. This approach mirrors the findings of Lawlor and Hanratty [18], who highlighted the effectiveness of personalized counseling in encouraging physical activity. Additionally, it aligns with the work of Harland et al. [19], which showed that tailored interventions integrated within routine care can effectively promote physical activity.

To achieve this, we adapted general recommendations to fit participants’ everyday lives. For example, we suggested using the stairs as a high-intensity exercise break, which has been shown to increase physical activity levels [20]. Despite the specific sample of participants, our experiment supports the conclusions of Kahn et al. [21], who found that an individualized approach to counseling is particularly effective for health behavior change. These findings are further reinforced by Aittasalo et al. [17], who emphasized the importance of tailoring interventions to individual contexts for greater effectiveness.

Consistent with our findings, a recent scoping review by Sui et al. [22] concluded that the association between subjective well-being and overall sedentary behavior is weak. The review also suggested that time spent in specific life domains, such as leisure screen time, may be more relevant to well-being than overall sedentary behavior. In our study, participants reported high baseline well-being scores, yet no significant association with sedentary behavior was found. This was particularly notable given their relatively high levels of physical activity, with the largest portion of sedentary time occurring during work hours. Based on these observations, one might speculate that sedentary behavior during work may either have a minimal detrimental effect or could be compensated for by the physical activity behavior during other parts of the day.

Despite clinically small yet statistically significant changes in physical activity, our longitudinal data revealed that these changes were insufficient to impact subjective well-being, as assessed using the WHO-5 scale. This contrasts with earlier studies that reported changes in well-being after interventions such as 4-week educational programs [23], 12-week online programs [24], or a 6-month sedentary behavior reduction intervention [25]. A key distinction of our study is its real-world approach: a single counseling session, as commonly used in workplace settings. By evaluating this low-intensity strategy, we critically examined whether such brief interventions provide measurable benefits. Nevertheless, this format also represents a limitation, as more intensive, multi-session interventions have shown stronger effects. Future studies should therefore incorporate scalable motivational components—such as the system by Ahmadi et al. [26]—to achieve more meaningful behavior change. The absence of an impact of sedentary behavior on well-being in our sample may be explained by the influence of other personal and work-related factors, such as job satisfaction, support from managers and colleagues, or personal life events, which are likely to play a more prominent role in determining subjective well-being.

Likewise, our data neither showed an association with sedentary behavior during baseline nor an intervention effect on self-perceived bodily pain. In line with our data, a systematic review published in 2010 analyzed possible triggers for the occurrence of low back pain, but could not confirm sitting during working hours as a trigger [27]. Furthermore, our physically active office workers reached rather low volumes of leisure sedentary time. In 2021, a meta-analysis examined the specific effects in different occupational groups and found an increased risk of back pain in office workers with a sedentary lifestyle [28]. Overall, the lack of an effect from a single counseling session highlights the need for more research on the long-term effects of sustained or multi-component interventions. While reducing sedentary behavior can benefit some aspects of health, its effects on subjective well-being and pain may be less pronounced than commonly assumed, especially in already physically active individuals.

Several limitations of this study should be considered when interpreting the results. First, the retrospective trial registration represents a limitation, as prospective registration is recommended to enhance transparency and reduce potential reporting bias. Second, physical activity and sedentary behavior were assessed via self-report, which is susceptible to recall and social desirability bias. However, both IPAQ and NPAQ are validated instruments with established reliability, and the counseling focused on concrete, easily remembered strategies, which may have improved the accuracy of participants’ self-reports. Finally, the intervention consisted of a single counseling session in a highly active sample, limiting the potential for further improvements and the generalizability of the findings to less active populations. A priori sample size calculation was not performed, as no comparable studies were available for this specific population, limiting the ability to estimate expected effect sizes. Consequently, the study may have been underpowered to detect small changes in physical activity or sedentary behavior.

## Conclusions

This study demonstrates that a single counseling session—despite being a commonly implemented workplace strategy—has only minimal effects on physical activity in already active office workers. While small reductions in workplace sedentary time and the introduction of active breaks were observed, overall physical activity and well-being did not change. More substantial effects are likely to require multi-session and multi-component interventions that combine individual behavior change strategies with supportive environmental and organizational measures.

## Supplementary Information

Supplementary Material 1.

## Data Availability

The datasets generated and/or analyzed during the current study are available from the corresponding author on reasonable request.

## Abbrevations

ANOVA: Analysis of variance
BMI: Body mass index
CONSORT: Consolidated standards of reporting trials
DKV: Deutsche Krankenversicherung
DRKS: German clinical trials register
IPAQ: International Physical Activity Questionnaire
MET: Metabolic equivalent of task
NHANES: National Health and Nutrition Examination Survey
NPAQ: Nordic Physical Activity Questionnaire
SF-36: 36-Item Short Form Health Survey
WHO: World Health Organization
WHO-5: World Health Organization-Five Well-Being Index
η²: Eta squared
