# Supplementary material for: Reducing sedentary behavior through workplace counseling: effects on activity, sitting breaks, and well-being – a randomized controlled trial
Source: Arch Public Health. 2026 Feb 28;84:67. doi: 10.1186/s13690-026-01867-6 (PMC13059149; doi:10.1186/s13690-026-01867-6)
Supplement: Supplementary file 1 — Supplementary Material 1. [file 13690_2026_1867_MOESM1_ESM.pdf]

Gesundheitsförderliche körperliche Aktivität

- Steigerung der Gesundheit
- Verbesserung des Schlafes
- Erhöhung der Lebensqualität
- Erhalt eines gesunden Gewichtes
- Reduzierung von Stress

Reduziert Risiko für

Diabetes Typ II	– 40%
Herzerkrankungen	– 35%
Stürze und Depressionen	– 30%
Rücken- und Gelenkschmerzen	– 25%
Krebs (Dickdarm/Brust)	– 20%

Noch heute beginnen: Es ist nie zu spät!

Auch Aktivität mit geringerer Dauer oder Häufigkeit hat schon positive Effekte

Alles zählt: Einkäufe tragen, zur Arbeit radeln, Hausputz

Ausdauer

Kombinationen sind möglich

Mindestens **150 Minuten / Woche** mit **moderater** Intensität

(Leicht verstärkte Atmung, Sprechen möglich, moderat anstrengend)

Schwimmen · *Zügiges Gehen* · *Radfahren*

oder

Mindestens **75 Minuten /** mit **hoher** Intensität

(Verstärkte Atmung, Sprechen erschwert, sehr anstrengend)

Joggen · *Sport* · *Treppen gehen*

Kraft

Kräftigung der Muskeln, Knochen und Gelenke

mindestens 2 Tage pro Woche

Fitness / Krafttraining · *Schwere Einkäufe tragen*

Job & Zuhause

Sitzende Zeit **reduzieren** oder **unterbrechen**

Ab 65 Jahren → **Schulung des Gleichgewichts**

Reduzierung des Sturzrisikos

Tanzen · *Bowling* · *Tai Chi*

Quelle: UK Chief Medical Officers' Physical Activity Guidelines 2019 / Goethe Universität, Abteilung Gesundheit und Leistung, PD Dr. Dr. Tobias Engeroff
